# Quality of life of patients with nonsuicidal self-injury: The role of suicidal ideation

**DOI:** 10.1192/j.eurpsy.2021.1575

**Published:** 2021-08-13

**Authors:** M. Zinchuk, G. Kustov, N. Voinova, E. Pashnin, R. Akzhigitov, A. Guekht

**Affiliations:** 1 Suicide Research And Prevention, Moscow Research and Clinical Center for Neuropsychiatry, Moscow, Russian Federation; 2 Mental And Neurological Disorders Department, Moscow Research and Clinical Center for Neuropsychiatry, Moscow, Russian Federation; 3 Department Of Neurology, Neurosurgery And Medical Genetics, Pirogov Russian National Research Medical University, Moscow, Russian Federation

**Keywords:** Suicide, Self-injurious thoughts and behaviors interview, NSSI, quality of life

## Abstract

**Introduction:**

Lower quality of life (QoL) scores are associated with suicidal behavior, both in the general population and in psychiatric patients. Nonsuicidal self-injury (NSSI) behavior is a public health concern because of its increasing prevalence and high risk of lifetime suicide attempt. Despite its significance QoL in patients with NSSI is underinvestigated. There is a gap in knowledge on the influence of suicidal ideation (SI) on certain domains and facets of QoL in patients with NSSI.

**Objectives:**

The study aimed to assess the impact of SI on QoL of patients with NSSI.

**Methods:**

We conducted a case-control study (1:3): 13 consecutive patients (11 female) with non-psychotic mental disorders and NSSI without lifetime SI were compared to 39 age and gender matched patients with NSSI and SI. All patients were evaluated by a psychiatrist, underwent Self-Injurious Thoughts and Behaviors Interview (Nock MK et al., 2007) and filled out the World Health Organization Quality of Life Assessment 100 (WHOQOL-100). Mann-Whitney and Fishers exact test were used as statistical methods.

**Results:**

The overall QoL (p=0.001) and the perception of life (p=0.005) were significantly higher in patients without SI. Patients with SI had a lower scores in psychological (p=0.002), social (p=0.036) and spiritual (p=0.005) domains as well as lower rates in energy (p<0.02); positive emotions (p<0.001); thinking, learning, memory and concentration (p=0.007); self-esteem (p=0.013); negative emotions (p=0.035); activities of daily living and participation (p=0.014) and opportunities for recreation/leisure facets (p=0.007).

**Conclusions:**

SI in patients with NSSI was found to be associated with worse QoL

